# Chloroform Fatalities.—III

**Published:** 1895-08-31

**Authors:** Benjamin Ward Richardson


### Chloroform Fatalities.—III.

By Sir Benjamin Ward Richardson, M.D., F.R.S.

As the remarks made in my previous papers seem still to attract considerable interest, it is worth while to dwell on two or three additional topics.

Much discussion has taken place as to the mode of death from chloroform vapour, and of one matter I am quite certain, namely, that the accident of death is much more common to the human animal than to animals of an inferior order, even the highest of the inferior animals—a fact which shows that the mental effect, the dread of the administration, is a very powerful agency towards dissolution amongst the human species of all classes, men, women, and children. I have had the opportunity during the past ten years of witnessing the influence of chloroform as a lethal agent on dogs anæsthetised in order to insure for them a painless death. I am able to speak from observation in at least five thousand examples, but I have never once known an instance in which a lower animal has died accidentally under chloroform as the human animal sometimes does. On the contrary, I have rather found it a difficulty to administer such large doses, by the lungs, as immediately to kill, and I am still looking out for an anæsthetic with a sufficient rapidity that shall replace chloroform in this respect.

From the effect just named I should be inclined to put down fear as one of the most determinate causes of fatality from chloroform. I have before me several instances in which it is impossible that chloroform, minus fear, could have been the direct cause of death, because sufficient of it was not administered to produce death. A lady in apparently good health went into the room of a friend of mine, an operating dentist, to have her teeth examined and attended to. It was found necessary to extract one or two teeth, and it was proposed that she should inhale chloroform for the purpose of preventing the pain. Everything was done to encourage her. The operator assured her, as was quite true, that he had never seen anything untoward in administration, promised that the least possible vapour should be given, and promised success for the removal pain; at last his arguments and promises prevailed. He put a drachm of chloroform into his inhaler and commenced to administer, on which the patient died I may say almost instantaneously, for forty minims of chloroform remained in the inhaler after death had become irrevocable. With the utmost distress at all times for the advice he had given, my friend still held that death was not attributable to the physiological action of chloroform on the body, and I believe he was strictly correct and that the event was what may be called a mental death. In fact that lady died because she was among the *morituri.* She would have died under a railway shock; on hearing sudden and bad news; nay, in a moment of great excitement from happy news itself; in fact from anything that would have affected the heart through the senses. People of this character died immediately before an operation in the days when anæsthetics were not dreamed of. I employ, in practice, for a disinfectant, a saturated solution of benzoic acid in chloroform, which I call benzoated chloroform. When I am engaged in practice and my hands come into contact with anything objectionable I wash them with water, dry them, and then put in the palms two or three drachms of this solution with which I cover the hands all over, and am, as far as the matter of chloroform is concerned, in quite as much danger from inhalation as the lady was whose case I have recorded. I have done this for years past; I have seen it done by others for years past; I have seen men making chloroform; I have seen them producing bichloride of methylene from chloroform; I have seen the bottles of chloroform intended for distribution and export prepared and filled, and I have tried minutely to ascertain if anybody ever died from accidentally inhaling the vapour yielded. I have never met with such an instance, and I do not believe anyone ever did.

The event of sudden death may occur upon inhalation of the bichloride of methylene; in truth, I know of it once taking place in that way. A young person was made to inhale methylene, and 40 drops were put into the inhaling apparatus. With one or two brief inhalations the patient died, but strictly it was no death from methylene in the manner of a toxical substance. It was fear that was the cause of death. This patient belonged, like the other, to the *morituri.* I once saw a man fall to his feet and nearly die on suddenly looking over the battlements of St. Albans Abbey; it was fear again that was at work; a peculiarly sensitive terror which abounds in certain natures, and which under the administration of the narcotic vapour is the element of danger. How this element of fear produces the sudden collapse I am unable to say. I never heard a reasonable explanation. It must be some direct impulse through the expanses of the nervous system telling directly upon an enfeebled heart, which it either checks in systole or too rapidly excites into systole, whereupon the organ is left, for a moment, empty of blood, and the syphon action is lost. In other words the column of blood is broken, probably both on the venous and arterial side, a change from which I believe there is no recovery. The general question is whether chloroform or the chlorides more than other anæsthetics become favourable to the deadly action of fear. It has generally been accepted that the majority of sudden deaths do arise under the action of chloroform and its allies, and many persons think, therefore, that ether, chloroform, ethylene, and nitric oxide are safer agents to administer even to the *morituri,* or those who are physically ready to die, as if the minds of those who received the narcotic were more composed and stronger when what was supposed to be a safer narcotic than chloroform was being administered. I can imagine in the case of a medical man who knew all the facts that the influence of knowledge in this respect might exert a directing and favourable influence, but it can scarcely be supposed that persons who have no knowledge at all or who do not know what anæsthetic is really being used can be stricken from the same cause.

